# Laparoscopic resection of a low-grade appendiceal mucinous neoplasm in a patient with autosomal dominant polycystic kidney disease: a case report

**DOI:** 10.1097/RC9.0000000000000953

**Published:** 2026-09-16

**Authors:** Masaya Aizawa, Shingo Tsujinaka, Yoh Kitamura, Yuuri Hatsuzawa, Tomoya Miura, Toru Nakano

**Affiliations:** Division of Gastroenterologic Surgery, Department of Surgery, Tohoku Medical and Pharmaceutical University, Sendai, Japan

**Keywords:** autosomal dominant polycystic kidney disease, case report, differential diagnosis, laparoscopic surgery, low-grade appendiceal mucinous neoplasm, pseudomyxoma peritonei

## Abstract

**Introduction and importance:**

Appendiceal mucinous neoplasms (AMNs) are rare, and preoperative diagnosis can be challenging, particularly when other abdominal cystic diseases coexist. Autosomal dominant polycystic kidney disease (ADPKD), which is characterized by multiple cystic formations, can complicate both differential diagnosis and surgical planning.

**Case presentation:**

A 77-year-old man with ADPKD and chronic renal failure was referred to our hospital following a positive fecal occult blood test during an evaluation for anemia. Colonoscopy revealed a large submucosal tumor-like elevation in the cecum, obscuring the appendiceal orifice. Non-contrast computed tomography showed multiple abdominal cysts and a low-attenuation lesion protruding into the cecal lumen with tubular extension outside the cecum. An AMN was suspected, although the potential for malignancy and pseudomyxoma peritonei could not be completely excluded. Despite concerns that the cysts might limit the operative view, pneumoperitoneum provided adequate visualization since the cysts were primarily retroperitoneal. Laparoscopic ileocecal resection with D2 lymph node dissection and primary ileocolic anastomosis was completed without tumor rupture. No ileostomy was created. The postoperative course was uneventful. Histopathological examination confirmed a low-grade AMN. No recurrence was observed 9 months postoperatively.

**Clinical discussion:**

This case underscores the challenge of differentiating AMNs from other cystic lesions in patients with ADPKD and suggests that cyst distribution may influence the surgical approach and exposure.

**Conclusion:**

Laparoscopic surgery may be a feasible option for patients with multiple abdominal cystic diseases when the cysts are predominantly located in the retroperitoneum.

## Introduction

Appendiceal mucinous neoplasms (AMNs) are relatively rare, yet their reported incidence has increased in recent years^[^1–3^]^, likely due to advances in diagnostic imaging and the widespread adoption of laparoscopic surgery, which enables thorough inspection of the peritoneal cavity^[^3,4^]^. However, definitive preoperative diagnosis can remain elusive, particularly when concomitant cystic diseases complicate the differential diagnosis.

HIGHLIGHTS

- Appendiceal mucinous neoplasms may mimic cystic lesions in patients with autosomal dominant polycystic kidney disease (ADPKD).
- ADPKD can complicate both preoperative differential diagnosis and surgical planning.
- The distribution of cysts may affect laparoscopic visibility and operative working space.
- Laparoscopic ileocecal resection was safely performed without tumor rupture.
- A predominantly retroperitoneal cyst distribution may support the feasibility of laparoscopic surgery.

Autosomal dominant polycystic kidney disease (ADPKD), the most common inherited kidney disorder, is characterized by the progressive development of multiple bilateral renal cysts, often accompanied by extrarenal manifestations. As cyst burden increases, renal function declines, with nearly half of affected patients progressing to end-stage renal disease by 60 years of age^[^5,6^]^.

We report the case of a low-grade appendiceal mucinous neoplasm (LAMN) in a patient with ADPKD. Preoperative imaging alone did not reliably differentiate the appendiceal lesion from other intra-abdominal cystic lesions or adequately assess its malignant potential. Furthermore, the multiple cysts associated with ADPKD were expected to limit the operative working space during laparoscopy, impair visualization, and necessitate modifications in port placement or patient positioning^[^7^]^. Despite these concerns, anticipated preoperatively, laparoscopic surgery was completed safely without tumor rupture. This case is reported in accordance with the Surgical Case Report (SCARE) guidelines^[^8^]^.

## Presentation of case

A 77-year-old man was referred to our hospital after a positive fecal occult blood test during an evaluation for anemia. His comorbidities included ADPKD, chronic renal failure, hypertension, and type 2 diabetes mellitus, and his medications included indapamide, doxazosin, azilsartan, azelnidipine, imidapril, benidipine, nifedipine, dapagliflozin, and insulin lispro.

His height was 160.0 cm, and his body weight was 62.2 kg, with a body mass index of 24.3 kg/m^2^. Physical examination revealed an irregularly contoured mass that was palpable from the right upper quadrant to the right flank, consistent with ADPKD. Laboratory findings were as follows: white blood cell count, 5000/µL; hemoglobin, 10.5 g/dL; platelet count, 163 000/µL; total bilirubin, 0.49 mg/dL; aspartate aminotransferase, 13 U/L; alanine aminotransferase, 6 U/L; blood urea nitrogen, 48 mg/dL; creatinine, 4.08 mg/dL; C-reactive protein, 0.59 mg/dL; carcinoembryonic antigen, 2.5 ng/mL; and carbohydrate antigen 19-9, 8.1 U/mL.

Colonoscopy revealed a large mass occupying most of the cecum. The mucosal surface was intact, and the lesion appeared as a submucosal tumor-like elevation covered with non-neoplastic mucosa, suggestive of either a submucosal tumor or extrinsic compression (Fig. 1A). The appendiceal orifice could not be identified. A contrast enema demonstrated a well-demarcated, round filling defect with smooth margins in the ileocecal region (Fig. 1B).

**Figure 1.**
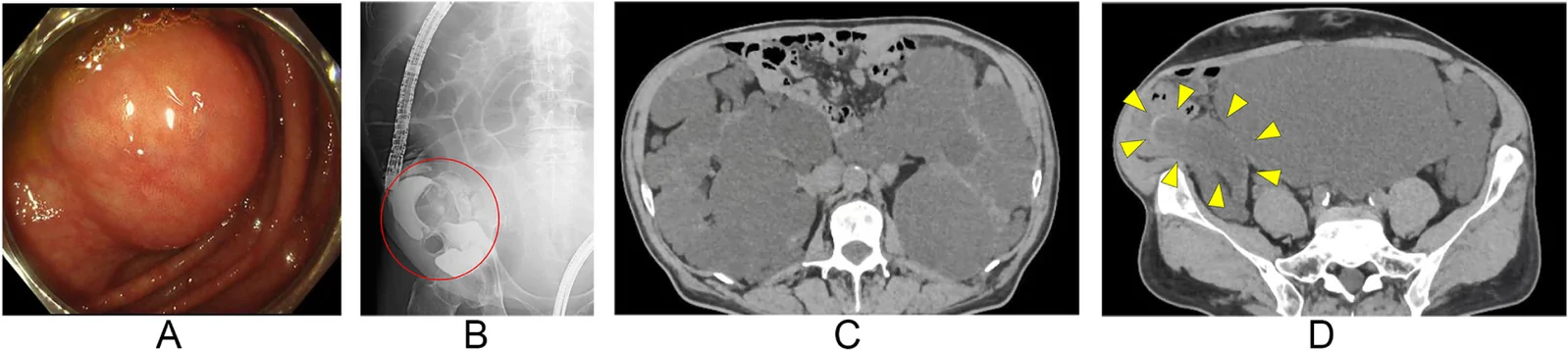
Preoperative imaging studies. (A) Colonoscopy shows a large mass occupying most of the cecum, appearing as a submucosal tumor-like elevation covered with non-neoplastic mucosa. (B) Contrast enema reveals a well-demarcated, round filling defect with smooth margins in the ileocecal region (red circle). (C) Non-contrast abdominal CT demonstrates multiple cysts throughout the abdomen, with ventral displacement of the intra-abdominal organs. (D) Non-contrast abdominal CT demonstrates a low-attenuation lesion protruding into the cecal lumen and extending outward in a tubular configuration beyond the cecum (yellow arrowheads).

Non-contrast abdominal computed tomography (CT) showed multiple cysts throughout the abdomen, with ventral displacement of the intra-abdominal organs (Fig. 1C). In the ileocecal region, a low-attenuation lesion protruding into the cecal lumen and extending in a tubular fashion outside the cecum was identified (Fig. 1D). Because of chronic renal failure, contrast-enhanced CT was not performed, limiting assessment of mural enhancement, wall characteristics, and the precise relationship of the lesion to the appendiceal base and cecum. Based on cecal continuity and tubular extra-cecal extension, an AMN was considered the most likely diagnosis, and laparoscopic resection was planned. However, preoperative malignant potential remained indeterminate, and differential diagnoses included ADPKD-related renal or hepatic cysts, appendiceal carcinoma, and pseudomyxoma peritonei (PMP).

Intraoperatively, adequate visualization was achieved after pneumoperitoneum was established, despite preoperative CT indicating that multiple cysts might obscure the operative view. The cysts were confined to the retroperitoneal space, displacing the surrounding anatomy (Fig. 2A). The appendix, located dorsal to the cecum, was markedly enlarged throughout its length, with the lesion extending to its base and protruding into the cecal lumen (Fig. 2B). Taken together, these findings supported an appendiceal origin and a mucinous neoplasm rather than an ADPKD-related cystic lesion, although gross inspection alone could not determine the malignant potential or exclude invasive appendiceal carcinoma. No mucinous ascites, peritoneal nodules or dissemination, serosal tumor exposure, or tumor rupture were observed. Therefore, laparoscopic ileocecal resection with D2 lymph node dissection was selected to secure margins, avoid rupture, and allow nodal staging. In this case, D2 lymph node dissection included removal of the pericolic and intermediate mesenteric lymph nodes within the ileocolic vascular territory, without apical lymph node dissection at the root of the ileocolic vessels. Bowel continuity was restored by extracorporeal primary ileocolic anastomosis, and no diverting ileostomy was created. The intraoperative findings and key steps of the surgical procedure are presented in Supplemental Digital Content Video S1, available at: https://links.lww.com/IJSCR/A163.

**Figure 2.**
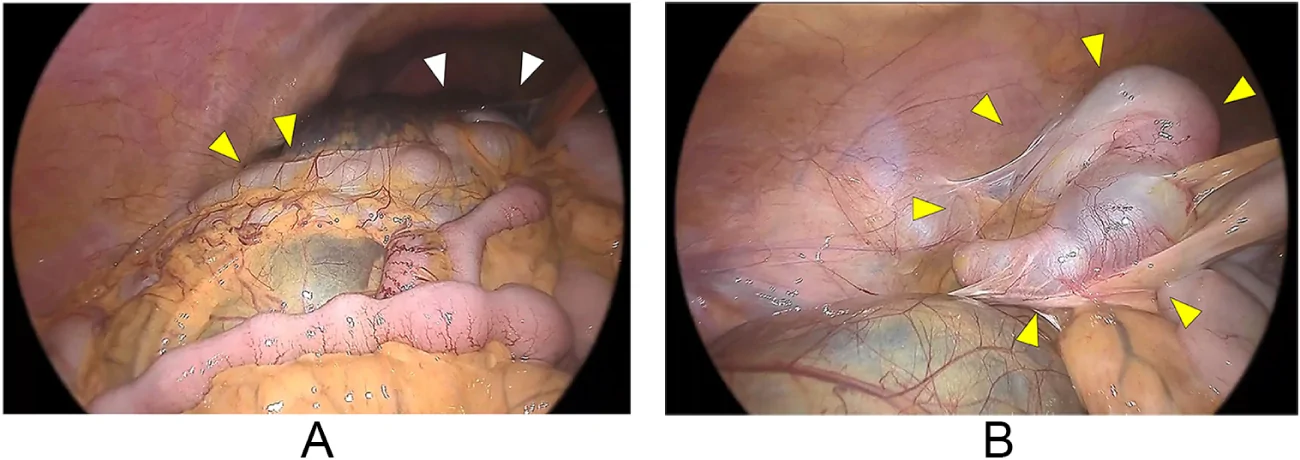
Intraoperative images. (A) The cysts are confined to the retroperitoneal space, resulting in displacement of the surrounding anatomy. The hepatic flexure (yellow arrowheads) is displaced away from the liver (white arrowheads). (B) The appendix is located dorsal to the cecum and is markedly enlarged throughout its length (yellow arrowheads).

The postoperative course was uneventful, and the patient was discharged on postoperative day 9. Macroscopic examination of the resected specimen revealed a tumor protruding into the cecal lumen, filled with gelatinous material, and accompanied by mucin-containing cystic structures within the appendiceal wall. No mural rupture or serosal tumor exposure was observed (Fig. 3A, B, and C). Resection margins were negative, and no lymph node metastasis was identified (0/12 harvested lymph nodes). Microscopically, the luminal surface was covered by papillary proliferation of tall columnar epithelial cells containing abundant intracytoplasmic mucin, without high-grade cytologic atypia, consistent with an LAMN (Fig. 3D). The tumor was confined to the appendiceal wall and staged as pTis (LAMN) N0. No recurrence was observed 9 months postoperatively.

**Figure 3.**
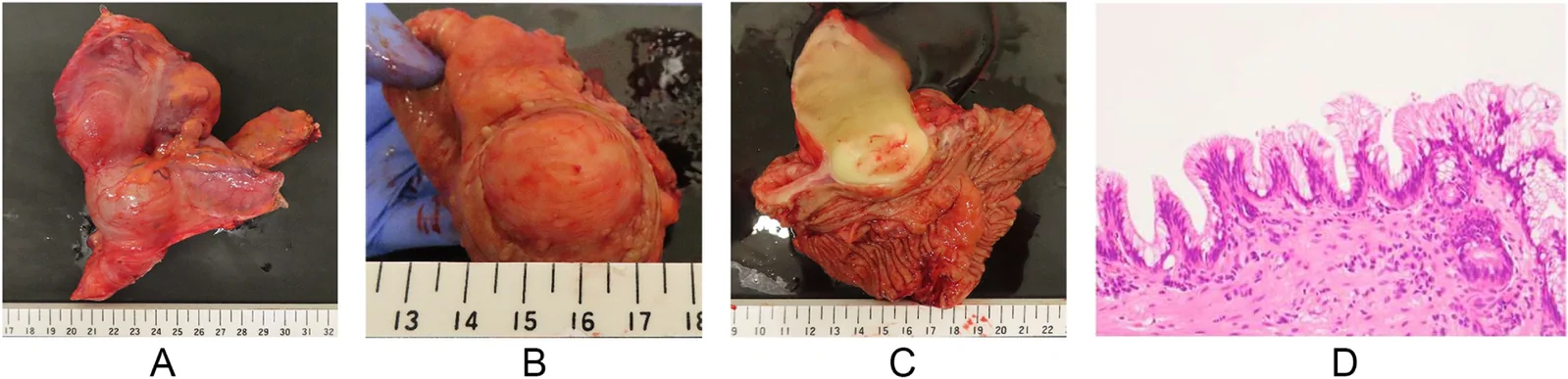
Macroscopic and microscopic findings of the resected specimen. (A) Gross appearance of the resected specimen shows no tumor exposure on the serosal surface. (B) Intraluminal view shows the tumor protruding into the cecal lumen. (C) Cross-sectional view shows that the cystic tumor contains gelatinous material. (D) Hematoxylin and eosin staining shows tall columnar epithelial cells with abundant intracytoplasmic mucin and without marked cytologic atypia, consistent with a low-grade appendiceal mucinous neoplasm (original magnification ×200).

## Discussion

Preoperative diagnosis of AMNs can be challenging, particularly when associated with concomitant ADPKD, which complicates the differential diagnosis^[^9^]^. In the present case, the primary differential diagnoses included cystic lesions arising from other organs, appendiceal carcinoma, and PMP.

Several reports indicate that AMN can mimic cystic lesions of other organs, including adnexal, renal, and hepatic cysts, with the definitive diagnosis often being made intraoperatively or pathologically^[^10–14^]^. In the present case, CT revealed a relationship between the appendiceal tumor and the cecum, along with a tubular extension outside the cecum, supporting AMN as the most likely diagnosis and allowing the exclusion of cystic lesions originating from other organs.

Differentiating LAMN from appendiceal carcinoma is clinically important. Yu *et al*^[^15^]^ described CT findings suggestive of LAMN, such as tumor morphology, intraluminal attenuation, wall characteristics, and enhancement patterns; however, these features lack specificity, making it difficult to reliably distinguish LAMN from invasive or high-grade disease.

Differentiation from PMP is also critical, as multiple cysts associated with ADPKD can resemble multiloculated cystic lesions on CT. Typical imaging characteristics of ADPKD include multiple well-defined round or oval cysts, with kidney enlargement and irregular contours as the disease progresses. In addition, multiple cysts may develop in organs other than the kidneys^[^5^]^. In the present case, cysts were primarily located in the kidneys and retroperitoneal space, with no evidence of intraperitoneal mucinous ascites or peritoneal deposits; therefore, PMP was considered unlikely. Nevertheless, it was difficult to completely exclude PMP based solely on preoperative imaging.

According to the American Society of Colon and Rectal Surgeons Clinical Practice Guidelines^[^1^]^, the surgical approach for AMNs varies based on malignant potential. For appendiceal carcinoma, lymph node metastasis is a concern, warranting ileocecal resection or more extensive colectomy with lymph node dissection, similar to the treatment for colon cancer. Conversely, for LAMN, there is no universal consensus, and lymph node metastasis is considered infrequent, regardless of tumor depth, potentially obviating the need for lymph node dissection^[^2^]^. The role of more extensive surgery has also been debated; right hemicolectomy may not confer a survival advantage in patients with mucinous appendiceal tumors accompanied by peritoneal seeding or PMP^[^16,17^]^, and radical appendectomy has been proposed as an alternative to right colon resection in selected patients with epithelial appendiceal neoplasms^[^18^]^. However, accurately assessing malignant potential or atypical cell distribution based solely on preoperative imaging remains difficult. Therefore, when managing AMNs, it is crucial to prevent tumor rupture and intraperitoneal dissemination, which may lead to PMP^[^1,3^]^. In the present case, invasive malignancy and PMP could not be completely ruled out, and the lesion protruded into the cecal lumen, while the appendiceal orifice could not be identified endoscopically. These findings led to the decision to perform laparoscopic ileocecal resection with D2 lymph node dissection, an oncologically less extensive procedure than right hemicolectomy.

When large hepatic or renal cysts are present, open surgery is often preferred. When laparoscopic approaches are attempted, limitations such as limited working space, compromised visualization, and necessary modifications (e.g., atypical port placement or changes in patient positioning) may arise.

Preoperative CT suggested that enlarged renal cysts might compromise the laparoscopic operative field. However, intraoperatively, the cysts were primarily confined to the retroperitoneal space, and pneumoperitoneum provided adequate working space and visualization. This indicates that the distribution of cysts, particularly whether they are predominantly retroperitoneal or involve intra-abdominal organs, may be an important consideration when selecting the surgical approach for patients with ADPKD.

This case report has several limitations. The lack of contrast-enhanced CT due to impaired renal function might have affected diagnostic accuracy. In addition, as this is a single case report involving a rare clinical scenario, the generalizability of the findings is limited. Further accumulation of cases is warranted.

## Conclusions

This case underscores the importance of preoperative imaging for differentiating multiple cystic lesions. As malignancy cannot always be excluded preoperatively, meticulous surgery is essential to prevent rupture and dissemination. When cysts are predominantly retroperitoneal, pneumoperitoneum may offer sufficient exposure, supporting laparoscopic surgery as a viable option for patients with multiple abdominal cystic disease.

## Data Availability

The data that support the findings of this study are available from the corresponding author upon reasonable request.
